# A Robust and Universal LC–MS/MS Method for Determination of *N*‐Nitrosodimethylamine in Pharmaceuticals Using a C30 Column

**DOI:** 10.1002/jssc.70465

**Published:** 2026-06-12

**Authors:** Byungchan An, Unyong Kim, Sumin Seo, Jiyu Kim, Chohee Jeong, Woojin Jeong, Eunjin Ko, Juhyeon Kim, Hyun‐Deok Cho, Sang Beom Han

**Affiliations:** ^1^ Department of Pharmaceutical Analysis College of Pharmacy Chung‐Ang University Dongjak‐gu Seoul Republic of Korea; ^2^ Division of Non‐Clinic Studies Korea Institute of Toxicology Daejeon Republic of Korea

**Keywords:** C30 (triacontyl) stationary phase, genotoxic impurity, LC–MS/MS, *N*‐nitrosodimethylamine, universal method

## Abstract

Since the 2018 valsartan recall, the genotoxic impurity *N‐*nitrosodimethylamine (NDMA) has been frequently detected in various pharmaceuticals. Matrix variability often complicates routine testing, requiring customized analyses. We developed a universal LC–MS/MS method enabling consistent NDMA detection across diverse pharmaceuticals. Separation utilized a C30 column (150 mm × 4.6 mm I.D., 5 µm), offering superior retention and shape selectivity for polar nitrosamines compared to conventional reversed phases. Unlike C18 or pentafluorophenyl phases, which often exhibit limited retention for NDMA, the C30 phase provides exceptional shape selectivity and enhanced hydrophobic interactions. This structural advantage, supplemented by its resistance to phase dewetting under highly aqueous conditions further ensures robust resolution between NDMA and complex drug matrices. The mobile phase consisted of 0.1% v/v formic acid in water and methanol under gradient elution at 1.0 mL/min. Detection used atmospheric pressure chemical ionization in positive ion mode. The method demonstrated a linear range of 2.0–100.0 ng/mL (*r* ^2^ > 0.999), with a limit of detection of 1.0 ng/mL and a limit of quantification of 2.0 ng/mL. Validation per International Council for Harmonisation (ICH) guidelines confirmed accuracy (87.7%–115.5%) and precision (coefficient of variation, CV ≤ 10.7%). Application to 30 diverse pharmaceutical products, including sartans and ranitidine, showed robust resolution (> 2.0) between the analyte and active pharmaceutical ingredients (APIs). Notably, NDMA was quantified in historical batches of ranitidine (164.83 ± 5.68 ng/mL), nizatidine (10.25 ± 0.52 ng/mL), and amitriptyline (2.28 ± 0.19 ng/mL). While the histamine type 2 receptor antagonists significantly exceeded the acceptable daily intake limit, the remaining 27 products showed no detectable NDMA. These findings highlight the method's effectiveness for real‐world surveillance and the critical risk of post‐manufacturing NDMA generation during prolonged storage. This universal C30‐based method provides a practical, reliable tool for routine screening, facilitating regulatory compliance and improved patient safety without drugspecific method development.

**Abbreviations**
ADI: acceptable daily intake
APCI: atmospheric pressure chemical ionization
API: active pharmaceutical ingredients
CV: coefficient of variation
DAD: diode array detector
ESI: electrospray ionization
IS: internal standard
LC–MS/MS: liquid chromatography‐tandem mass spectrometry
NDMA: *N*‐nitrosodimethylamine
PFP: pentafluorophenyl
RPLC: reversed phase liquid chromatography
SIL‐IS: stable isotope‐labeled internal standard

## 1 Introduction

In July 2018, *N‐*nitrosodimethylamine (NDMA; *M*_r_ 74.08) contamination was first identified in valsartan drug substances manufactured by Zhejiang Huahai Pharmaceutical. Classified as a Group 2A carcinogen by the International Agency for Research on Cancer (IARC), NDMA is characterized by its potent mutagenic and carcinogenic potential [1, 2]. Since this initial report, NDMA has been consistently detected in various other therapeutic classes, notably ranitidine and metformin [1, 3]. Consequently, global regulatory agencies have established a strict acceptable daily intake (ADI) limit of 96 ng/day (based on a 50–60 kg adult) and have initiated recalls for products exceeding this threshold [2, 4, 5]. The US Food and Drug Administration (FDA) guidelines, “Control of Nitrosamine Impurities in Human Drugs,” mandate rigorous monitoring to ensure patient safety [2].

Nitrosamines typically form via acid‐catalyzed nitrosation, where secondary or tertiary amines react with nitrosating agents such as nitrite ions (NO_2_^−^) [6]. Beyond direct synthesis, NDMA can emerge through complex pathways, including interactions with residual amines in excipients or through thermal degradation of the active pharmaceutical ingredient (API) [7, 8, 9, 10]. As highlighted in a recent review by Manchuri et al. (2024), the severe health risks associated with these impurities have prompted intensified scrutiny from the FDA and European Medicines Agency (EMA), necessitating advanced analytical platforms capable of trace‐level quantification in increasingly complex matrices [11]. Given these concerns, pharmaceutical companies are actively investigating formation mechanisms and implementing preventive strategies. Consequently, accurate and reliable quantification of NDMA in both APIs and drug products is essential to ensure pharmaceutical safety and quality.

Therefore, developing sensitive and selective analytical methods to detect and quantify NDMA in APIs and drug formulations is crucial. Mass spectrometry (MS) coupled with liquid chromatography (LC) or gas chromatography (GC) remains the primary technique, providing the sensitivity and selectivity required by global regulatory agencies [9, 10, 11, 12, 13, 14, 15]. Recent literature has highlighted the critical role of sophisticated MS techniques in identifying probable carcinogens across diverse matrices. For example, highly sensitive GC‐MS platforms have been successfully deployed for the trace‐level determination of 1,4‐dioxane in cosmetic products [16], as well as for the selective quantification of NDMA [17]. However, given the typically low NDMA concentrations in samples, efficient analyte ionization is critical, as is achieving adequate resolution between the analyte and drug substance. Notably, LC is preferred over GC because high temperatures in GC injection ports can thermally induce artifactual NDMA formation, particularly in ranitidine samples [10, 18]. In GC/MS analysis, thermal stress can degrade ranitidine into dimethylamine and nitrite, which subsequently react in situ to form NDMA [5, 10].

While NDMA remains the target analyte, the diverse physicochemical properties of APIs often require drug‐specific and resource‐intensive analytical method development. Conventional reversed‐phase methods using C18 and pentafluorophenyl (PFP) columns often exhibit limited retentivity for NDMA due to its high polarity and small molecular size, leading to co‐elution with APIs. Therefore, a universal, adaptable analytical method is essential to accommodate this variability. Such a method would improve efficiency, simplify method transfer, reduce development burden, and enhance regulatory compliance.

To address the limitations of conventional reversed phases, we strategically employed a C30 (triacontyl) stationary phase. Unlike standard C18 phases, the C30 phase features significantly longer and more densely packed alkyl chains, creating a highly ordered, thick, and rigid hydrophobic layer [19]. This unique structural configuration confers exceptional shape selectivity (steric recognition), which is highly advantageous for discriminating small, polar molecules like NDMA from massive, structurally diverse API matrices. Furthermore, the extended alkyl chains of the C30 phase are highly resistant to hydrophobic collapse (phase dewetting) under the highly aqueous mobile phase conditions typically required to retain polar nitrosamines, thereby ensuring consistent and reproducible retention [19]. Ultimately, this synergistic combination of shape selectivity and robust structural stability eliminates the need for drug‐specific optimizations, serving as the fundamental basis for our universal analytical platform. By leveraging these superior chromatographic properties, this study aimed to develop and validate a universally applicable LC–APCI–MS/MS method for the sensitive and selective detection of trace NDMA across diverse pharmaceutical products. We optimized chromatographic parameters, including the stationary phase, mobile phase, and instrument settings, to achieve adequate resolution between NDMA and drug substances, thereby minimizing matrix effects. The method was validated using 30 diverse pharmaceutical products, including ranitidine, nizatidine, various sartans (valsartan, losartan, olmesartan, irbesartan, candesartan, and fimasartan), and other NDMA‐prone drugs such as tramadol hydrochloride, diltiazem, doxylamine succinate, rivastigmine tartrate, metoclopramide, entacapone, prednisolone, clarithromycin, chlorpheniramine, chlorpromazine hydrochloride, imipramine hydrochloride, amitriptyline hydrochloride, desvenlafaxine, and dutasteride.

## 2 Materials and Methods

### 2.1 Reagents and Materials

A standard stock solution of NDMA (200 mg/L) in methanol and the internal standard (IS), NDMA‐d_6,_ were purchased from Sigma‐Aldrich (St. Louis, MO, USA). HPLC and LC–MS grade water, methanol, and acetonitrile were obtained from Thermo Fisher Scientific (Fair Lawn, NJ, USA). Mobile phase additives, including LC–MS grade ammonium acetate, ammonium formate, acetic acid, and formic acid, were also sourced from Thermo Fischer Scientific. Syringe filters (0.20 µm polytetrafluoroethylene [PTFE] membrane) were purchased from Whatman (Maidstone, UK).

### 2.2 Pharmaceutical Products

**FIGURE 1:**
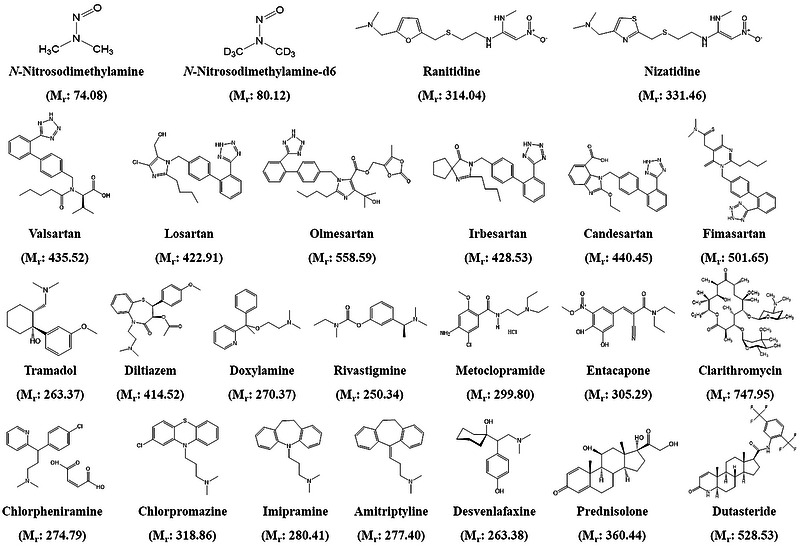
Chemical structures and molecular masses (*M*_r_) of *N‐*nitrosodimethylamine (NDMA), internal standard (NDMA‐d6), and 22 active pharmaceutical ingredients (APIs) investigated in this study. The molecular mass (*M*_r_) is indicated in parentheses for each compound.

The following drug substances were generously provided by pharmaceutical manufacturers: ranitidine hydrochloride, nizatidine, valsartan, losartan, olmesartan, irbesartan, candesartan, and fimasartan. The following drug products were purchased from CD Pharms (Seoul, South Korea): tramadol hydrochloride, diltiazem, doxylamine succinate, rivastigmine tartrate, metoclopramide, entacapone, prednisolone, clarithromycin, chlorpheniramine, chlorpromazine hydrochloride, imipramine hydrochloride, amitriptyline hydrochloride, desvenlafaxine, and dutasteride. Figure 1 illustrates the chemical structures and molecular masses of NDMA, NDMA‐d_6_, and the target APIs.

### 2.3 Instruments and Equipment

NDMA quantification was performed using an Agilent 1290 Infinity LC system (Santa Clara, CA, USA) coupled to an Agilent 6490 triple quadrupole mass spectrometer equipped with an atmospheric pressure chemical ionization (APCI) source. Data acquisition and processing were conducted using MassHunter Workstation software (Ver. B.06.00, Agilent Technologies). An Agilent 1260 Infinity LC system with a diode array detector (DAD) was utilized to validate chromatographic conditions and ensure adequate resolution between NDMA and APIs. Sample preparation involved using a Vortex‐Genie 2 mixer (Scientific Industries, Bohemia, NY, USA), a Green SSeriker shaker (Vision Scientific, Daejeon, South Korea), and a Combi R515 centrifuge (Hanil Science Industrial, Incheon, South Korea).

### 2.4 Sample Preparation

Each drug product was pulverized, and an amount equivalent to the maximum daily dose was accurately weighed into a 10 or 20 mL volumetric flask. A specific volume (5 or 10 mL) of the extraction solvent (water/methanol, 20:80, v/v) was added. For the tramadol/acetaminophen fixed‐dose combination, 100% methanol was used as the extraction solvent. The mixture was shaken for 30 s, then spiked with working standard solutions of analyte and IS. The flask was filled to volume with the extraction solvent, transferred to a 50 mL conical tube, and mechanically shaken for 30 min. Following centrifugation at 3500 × *g* for 10 min, the supernatant was filtered through a 0.20 µm PTFE syringe filter. A 10 µL aliquot of the filtrate was injected into the LC–MS/MS system.

### 2.5 LC–MS/MS Conditions

Chromatographic separation was achieved on a Develosil RPAQUEOUS‐AR C30 column (150 mm × 4.6 mm I.D., 5 µm; Nomura Chemical Co. Aichi, Japan). The mobile phase consisted of (A) 0.1% v/v formic acid in water and (B) 0.1% v/v formic acid in methanol, delivered at a flow rate of 1.0 mL/min. The gradient program was as follows: 0.0–4.0 min, 5% Solvent B; 4.0–6.0 min, linear increase to 90% Solvent B; 6.0–11.0 min, maintained at 90% Solvent B; and 11.0–18.0 min, re‐equilibrated at 5% Solvent B. The column temperature was maintained at 30°C. To prevent mass spectrometer contamination from high API concentrations, a divert valve was programmed to direct the flow to the detector only during the elution of the analyte and IS (2.5–4.5 min), diverting to waste at all other times (0.0–2.5 and 4.5–18.0 min). Uracil was injected as an unretained marker to determine the column void time (*t*_0_) for the calculation of the retention factor (*k*). MS analysis was performed in multiple reaction monitoring (MRM) mode using positive APCI. The optimized source parameters were: gas temperature, 200°C; vaporizer temperature, 375°C; gas flow rate, 15 L/min; nebulizer pressure, 30 psi; capillary voltage, 4500 V; and corona current, 8 µA. Nitrogen gas, generated from liquid nitrogen, served as both the nebulizer and collision gas. Quantification was performed using the following MRM transitions: *m/z* 75.0 → 43.0 for NDMA and *m/z* 81.0 → 46.0 for the IS.

### 2.6 Method Validation

The analytical method was validated in accordance with the International Council for Harmonisation (ICH) guideline Q2(R2), “Guideline on Validation of Analytical Procedures” [20]. Key validation parameters included specificity, linearity, accuracy, precision, limit of detection (LOD), limit of quantification (LOQ), robustness, and system suitability.

Specificity was assessed by analyzing blank samples, standard solutions, and actual samples to identify potential matrix interferences. Linearity was established using linear regression analysis of calibration curves (analyte/IS peak area ratio) across at least six concentration levels, ranging from the LOQ to 50‐fold the LOQ (*R* ^2^ ≥ 0.990). Accuracy was determined by calculating the percent recovery from samples spiked at low, medium, and high concentration levels. Precision was evaluated as the coefficient of variation (CV) of replicate measurements; intra‐day precision involved five replicate measurements per concentration, while inter‐day precision was assessed over five consecutive days. The LOD and LOQ were defined as the concentrations yielding a signal‐to‐noise (S/N) ratio on the chromatogram greater than 3 and 10, respectively. Robustness was examined by monitoring peak area variations under minor modifications in flow rate and column temperature (±10%). System suitability was confirmed by assessing the repeatability of the analyte‐to‐IS peak ratio from six replicate injections of the standard solution at the LOQ level.

## 3 Results and Discussion

### 3.1 Challenges in Analyzing Trace NDMA in Pharmaceuticals

NDMA is potent genotoxin, requiring rigorous monitoring at trace levels (ng/mL level range). While sample pre‐concentration is a standard procedure for trace analysis, the low molecular mass (74.1 g/mol) and volatility of NDMA (boiling point: 151°C) pose significant challenges [21]. Substantial analyte loss has been reported even under mild nitrogen evaporation at 40°C, leading to poor recovery rates. Consequently, an alternative strategy to circumvent the concentration step is the “dilute‐and‐shoot” approach, which involves dissolving a larger sample mass in a minimal volume of solvent to maintain analyte detectability [22, 23]. However, this method inevitably introduces massive excess of the drug substance (API) into the LC–MS/MS system, creating a challenging analytical environment characterized by a wide dynamic range as well as increasing the risk of severe system contamination.

In this context, the chromatographic elution order is a decisive factor for accurate quantification. As the concentration ratio of API to NDMA often exceeds 10^6^ [1, 5, 6, 13, 18, 19, 24], it is critical that trace impurity elutes prior to the major API component [25, 26]. If NDMA were to elute after or typically co‐elute with the high‐concentration API, its signal would likely be obscured by the severe peak tailing or suppressed due to ion suppression effects caused by the matrix overload. Therefore, ensuring the early elution of NDMA is essential to secure a clean baseline free from interference, thereby guaranteeing the reliability and sensitivity of the quantification. While this separation strategy also facilitates the use of a divert valve to protect the mass spectrometer, the primary analytical imperative is to isolate the trace analyte from the overwhelming signal of the major component.

However, achieving this ideal elution profile is challenging with conventional reversed‐phase liquid chromatography (RPLC) using C18 stationary phases. Due to the high polarity (log *P_ow_* = −0.57) and small hydrodynamic volume of NDMA, its interaction with standard octadecylsilane chains is weak, often resulting in insufficient retention or co‐elution with polar APIs near the solvent front. This lack of retentivity limits the ability to manipulate selectivity (*α*) effectively. Consequently, to ensure the consistent early elution and separation of NDMA from diverse APIs without extensive method redevelopment, a stationary phase offering enhanced shape selectivity and hydrophobic interaction—such as the C30 phase—is required.

### 3.2 Optimization of a Universal Reversed‐Phase HPLC Column

Achieving sufficient retention of NDMA in RPLC is challenging due to its small molecular size and high hydrophilicity. While increasing the water content of the mobile phase is a standard strategy to enhance retention, it typically has limited efficacy for NDMA on conventional hydrophobic stationary phases due to insufficient hydrophobic interaction and poor thermodynamic partitioning. Previous studies have employed various column chemistries, including PFP and biphenyl phases, to enhance selective retention via π–π interactions with the nitroso group of NDMA [27].

In this study, we evaluated nine stationary phases to optimize NDMA retention: four C18 columns and one each of C8, PFP, phenyl‐hexyl, cyano (CN), and C30. The retention times (*t_R_*) and retention factors (*k*) were determined for each column. Among the tested columns, five (Cadenza CX‐C18, Gemini C18, Eclipse XDB‐C18, phenyl‐hexyl, and C30) exhibited retention factors near or above 1.0 (Table 1). Notably, retention behavior varied significantly even among C18 stationary phases, depending on the manufacturer. For instance, the retention factor of the Ascentis Express column was approximately half that of other C18 columns, indicating that NDMA retention is critically influenced by stationary phase characteristics, such as carbon load, surface area, and silica end‐capping chemistry.

**TABLE 1:** Chromatographic retention behavior of *N*‐nitrosodimethylamine (NDMA) across various stationary phases. The table compares the retention time (min) and retention factor (*k*) of NDMA using nine different commercially available analytical columns, encompassing C18, C8, pentafluorophenyl (F5), phenyl‐hexyl, cyano, and C30 stationary phases. This extensive screening was conducted to evaluate the intrinsic retention capability of each phase for the highly polar NDMA molecule. The results highlight the distinctive retention properties of the C30 phase (Develosil RPAQUEOUS‐AR), which was ultimately selected for the proposed method due to its optimal shape selectivity and superior capacity to resolve NDMA from massive active pharmaceutical ingredient (API) peaks.

Stationary phase	Column	Retention time (min)	Retention factor (*k*)
**C_18_**	Cadenza CX‐C_18_ (150 mm × 4.6 mm, 3.0 µm)	3.6	1.1
	Phenomenex Gemini 5u C_18_ (150 mm × 4.6 mm, 5 µm)	3.7	1.1
	Agilent Eclipse XDB‐C_18_ (150 mm × 4.6 mm, 3.5 µm)	3.3	0.9
	Supelco Ascentis Express C_18_ (150 mm × 4.6 mm, 2.7 µm)	2.5	0.4
**C_8_**	Waters XTerra RP C_8_ (150 mm × 4.6 mm, 3.5 µm)	2.3	0.3
**Pentafluorophenyl**	Phenomenex Kinetex F5 (100 mm × 3.0 mm, 2.6 µm)	1.5	0.8
**Phenyl‐hexyl**	Phenomenex Luna phenyl‐hexyl (150 mm × 4.6 mm, 5 µm)	4.2	1.4
**Cyano**	Phenomenex Luna 3u CN (150 mm × 2.0 mm, 3 µm)	3.1	0.9
**C_30_**	Develosil RPAQUEOUS‐AR (150 mm × 4.6 mm, 5 µm)	3.5	1.0

Abbreviation: NDMA, *N*‐nitrosodimethylamine.

**FIGURE 2:**
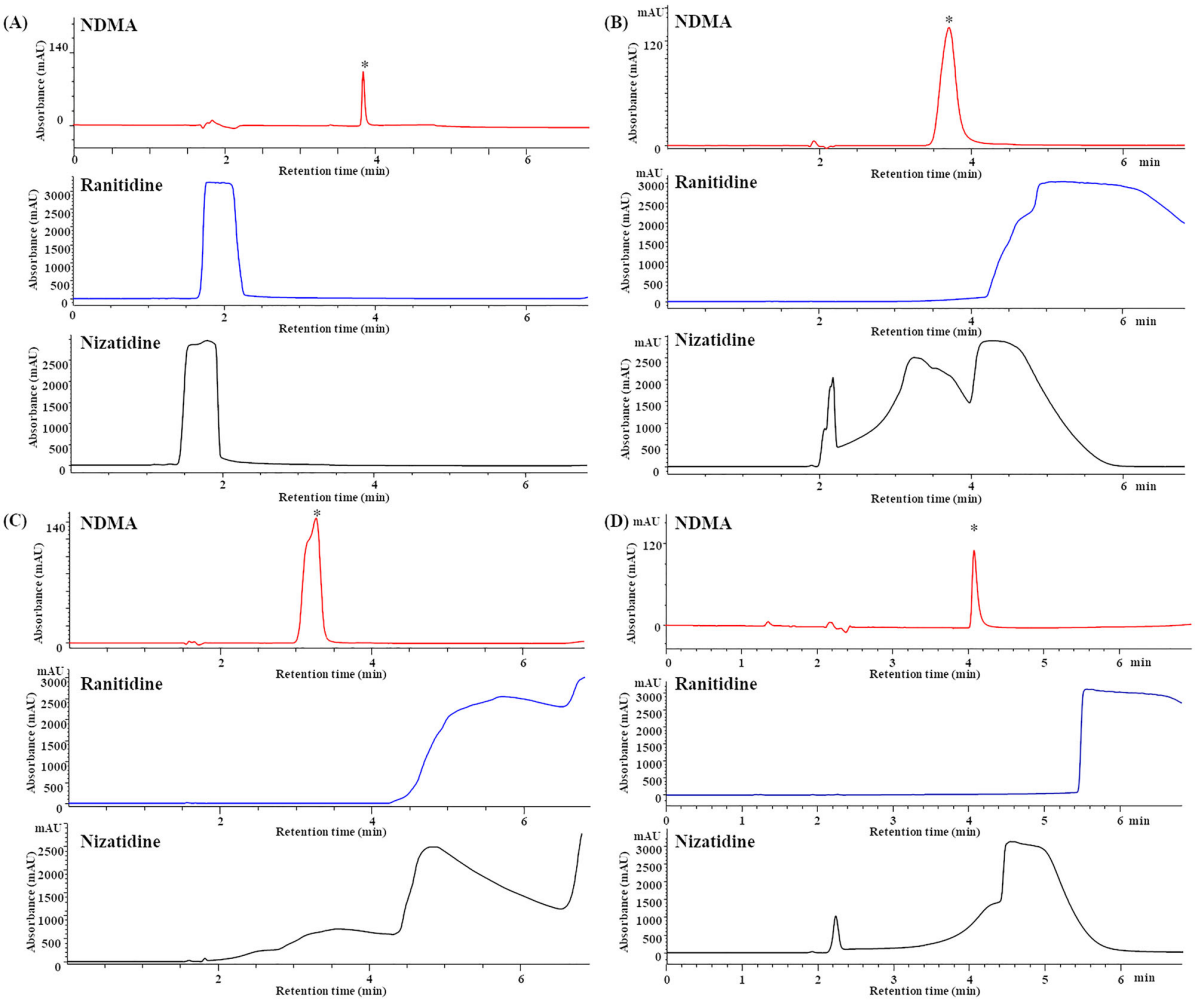
HPLC–DAD chromatograms (254 nm) demonstrating the retention profiles of NDMA (10 µg/mL, top red trace, marked with an asterisk *), ranitidine (30 mg/mL, middle blue trace), and nizatidine (15 mg/mL, bottom black trace) using various reversed‐phase columns: (A) Cadenza CX‐C18 (150 mm × 4.6 mm, 3 µm), (B) Gemini C18 (150 mm × 4.6 mm, 3 µm), (C) Eclipse XDB‐C18 (150 mm × 4.6 mm, 3.5 µm), and (D) Zorbax Eclipse Plus Phenyl‐Hexyl (150 mm × 4.6 mm, 3.5 µm). The stacked individual chromatograms illustrate that conventional C18 and phenyl‐hexyl columns result in severe peak overloading and broadening of the APIs, leading to direct co‐elution with the NDMA peak. The Cadenza column (A) shows partial separation, but the extensive tailing of the APIs extends into the NDMA retention window. Chromatographic conditions: mobile phase A: 0.1% v/v formic acid in water; mobile phase B: 0.1% v/v formic acid in methanol; flow rate: 1.0 mL/min; column temperature: 30°C; injection volume: 10 µL.

Subsequently, the five selected columns were assessed for their ability to separate NDMA from the most challenging APIs, specifically ranitidine and nizatidine. Although the Cadenza CX‐C18 column provided reasonable retention for NDMA, the massive API peak eluted earlier (approximately 2 min prior). Critically, the extensive tailing of the high‐concentration API peak extended into the retention window of NDMA (Figure 2A), leading to co‐elution. This overlap not only compromises quantification but also increases the risk of ion source or mass analyzer contamination, and carryover during repeated analyses.

In contrast, the Gemini C18, XDB‐C18, and phenyl‐hexyl columns failed to maintain chromatographic integrity under high sample load conditions (Figure 2B–D). Severe mass overload resulted in gross peak distortion, particularly for nizatidine (red trace). Instead of eluting as a discrete band, nizatidine exhibited a broad, multi‐modal elution profile extending from 2 to 7.5 min. This broadening heavily overlapped with the retention window of NDMA (*t_R_* ≈ 3.5–4.0 min). Although MS/MS offers mass selectivity, such extensive co‐elution with a massive excess of API inevitably induces significant matrix effects, particularly ion suppression. This phenomenon compromises the accuracy and reproducibility of quantification and drastically increases the risk of system contamination.

**FIGURE 3:**
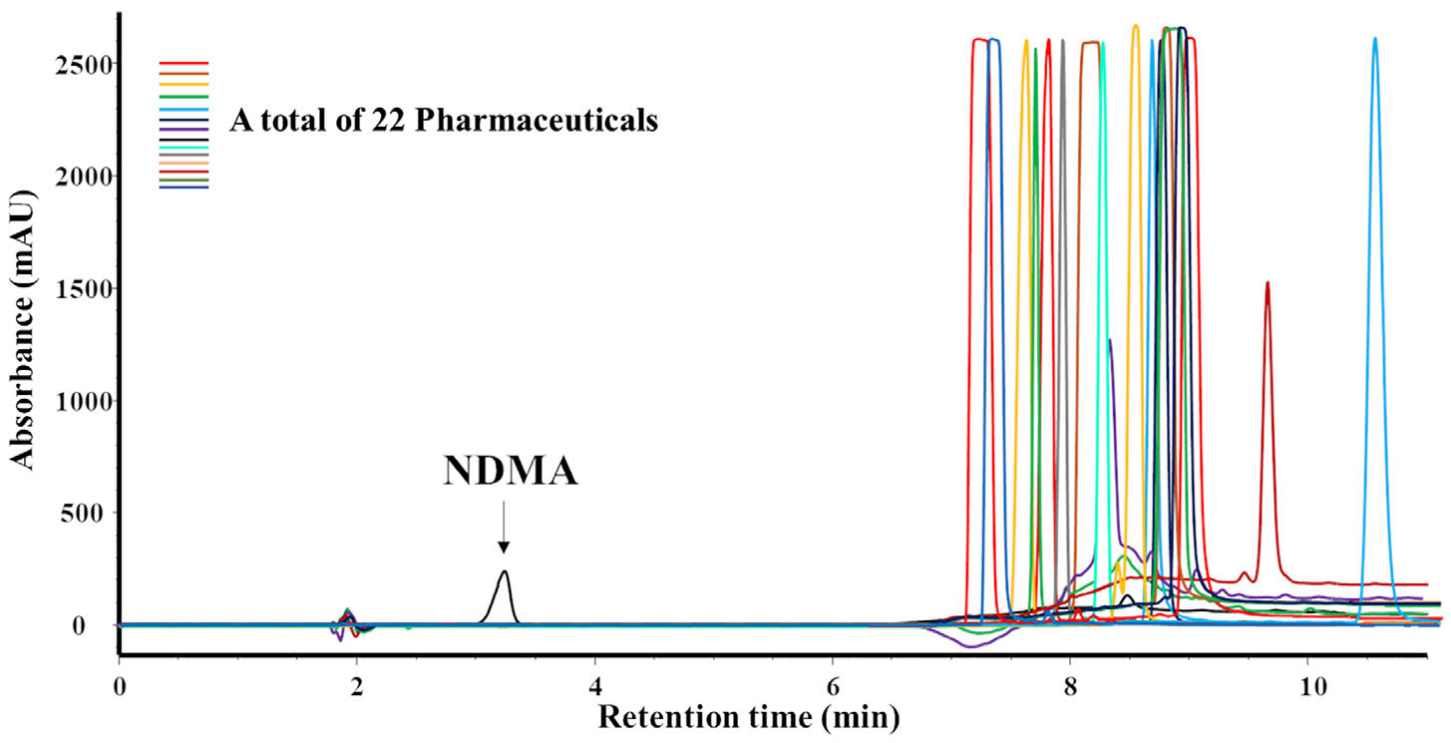
HPLC–DAD chromatograms obtained using the Develosil RPAQUEOUS‐AR C30 column (150 mm × 4.6 mm, 5 µm), demonstrating the separation of NDMA (50 ng/mL) from a mixture of 22 pharmaceutical products (1 mg/mL). The C30 stationary phase provides superior shape selectivity and retention, allowing the NDMA peak (retention time approx. 3.2 min) to be well‐resolved (*R*_s_ > 2.0) from the high‐concentration API peaks eluting later. Chromatographic conditions: mobile phase A: 0.1% v/v formic acid in water; mobile phase B: 0.1% v/v formic acid in methanol; gradient elution: 5% B (0–4 min), 5%–90% B (4–6 min), 90% B (6–11 min), 5% B (11–18 min); flow rate: 1.0 mL/min; column temperature: 30°C; detection: 254 nm.

The C30 stationary phase provided adequate NDMA retention and effectively resolved it from ranitidine, nizatidine, sartans, and other pharmaceuticals (Figure 3). The C30 phase exhibits significantly greater lipophilicity than conventional C18 phases, thereby enhancing the retention of moderately nonpolar compounds under identical chromatographic conditions [28, 29]. Additionally, its structural ordering confers superior shape selectivity, allowing for better discrimination based on molecular geometry [28, 30, 31, 32]. Furthermore, the C30 stationary phase offers more consistent retention and higher reproducibility in highly aqueous mobile phases compared to C18 phases [29, 33]. Literature suggests that the long alkyl chains of C30 phase prevent the hydrophobic collapse (phase dewetting), ensuring that the aqueous mobile phase maintains access to the mesopores of packing material [33].

The developed method demonstrated exceptional robustness across 22 different APIs, encompassing a broad spectrum of physicochemical properties, including a wide range of Log *P* values and ionization states (*p*K*_a_*). The C30 stationary phase, featuring long triacontyl chains, forms a thicker hydrophobic layer than standard C18 phases [28, 30, 32]. This structural configuration significantly enhances molecular shape selectivity and dispersive interactions. Consequently, even in presence of highly lipophilic APIs, the polar NDMA (Log *P* ≈ −0.57) is consistently retained, eluting at a stable retention time of approximately 3.8 min. This ensures effective separation from the drug matrix and minimizes co‐elution, confirming the C30 column's superiority as a universal platform for nitrosamine analysis in diverse pharmaceutical formulations.

### 3.3 Optimizing APCI‐MS/MS: Addressing Matrix Effects and Mobile Phase Selection

While electrospray ionization (ESI) is commonly used interface in LC–MS/MS for analyzing organic compounds, it is highly susceptible to matrix effect [34, 35]. This susceptibility arises because ESI relies on the ejection of ions from charged droplets during solvent evaporation. When co‐eluting matrix components are present, they compete with the target analyte for the limited charge sites on the droplet surface, leading to significant ion suppression [35]. In contrast, APCI relies on gas‐phase ion‐molecule reactions initiated by a corona discharge. Since ionization occurs after the solvent has fully evaporated, APCI is inherently less prone to the competition for charge observed in the ESI droplet phase [35]. Therefore, APCI is preferred for detecting trace amounts of small, non‐polar molecules like NDMA in complex pharmaceutical matrices. Consequently, this study employed positive mode APCI to maximize sensitivity and robustness.

**FIGURE 4:**
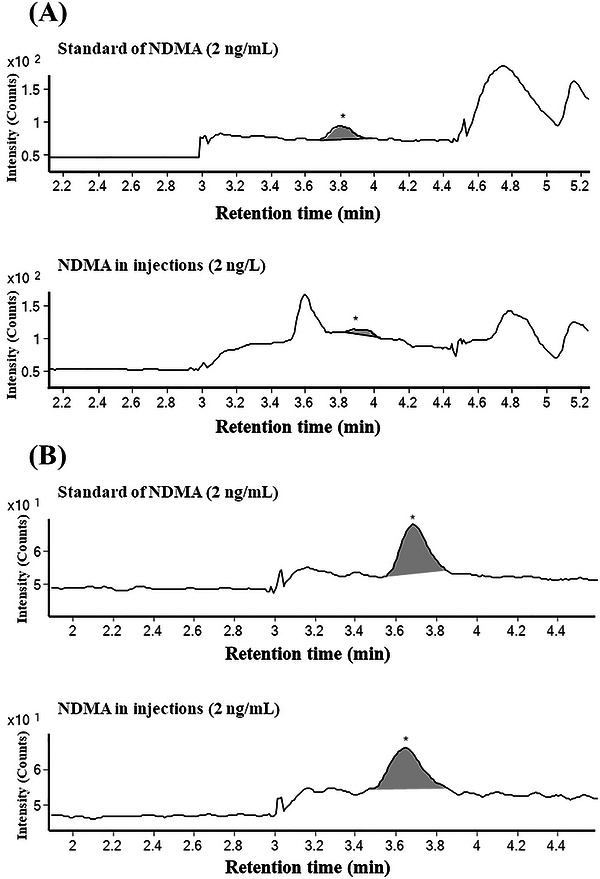
LC–APCI–MS/MS multiple reaction monitoring (MRM) chromatograms comparing matrix effects on NDMA detection. (A) Analysis using the Cadenza CX‐C18 column, showing significant ion suppression in the NDMA‐spiked sample compared to the standard solution due to co‐eluting excipients. (B) Analysis using the C30 column, showing consistent peak area and shape for both the standard and the spiked sample, indicating effective separation from matrix interferents. MS conditions: MRM transition *m*/*z* 75.0 → 43.0 for NDMA; APCI positive ion mode. NDMA concentration: 2 ng/mL.

Despite the inherent robustness of APCI, matrix effects derived from pharmaceutical excipients remained a challenge depending on the chromatographic separation. Notably, although the Cadenza CX‐C18 column provided adequate NDMA retention and peak symmetry of APIs, significant ion suppression was observed (Figure 4A). The peak area of NDMA in spiked samples was markedly lower than in standard solutions, indicating that unidentified excipients co‐eluted with the analyte, competing for charge in the source. This interference, likely due to the lack of extensive sample cleanup in the “dilute‐and‐shoot” method, compromised quantitative accuracy. Conversely, the C30 column (Figure 4B) exhibited no significant ion suppression across various formulations (tablets, injections, or soft capsules). Although specific interfering peaks were not visually distinct in the MRM channels, the consistent recovery and stable baseline confirm that the C30 phase effectively resolves NDMA from suppressing excipients, leveraging its superior shape selectivity.

Following column selection, mobile phase composition was optimized to enhance APCI sensitivity (Figure S1). A 0.1% v/v formic acid solution was selected as the aqueous phase for optimal peak shape and sensitivity for NDMA. For organic modifier, methanol provided superior sensitivity and retention stability compared to acetonitrile. This is attributed to the thermodynamics of APCI ionization; acetonitrile possesses a higher proton affinity (779 kJ/mol) than methanol (754 kJ/mol) and water (691 kJ/mol) [36, 37]. In positive APCI mode, solvent molecules with high proton affinity can effectively sequester protons, thereby suppressing the protonation of the target analyte [36]. Methanol, having a lower proton affinity, facilitates more efficient proton transfer to NDMA, resulting in higher response factors. Therefore, 0.1% v/v formic acid in both water and methanol was adopted.

Finally, the volatility of NDMA precludes evaporative concentration, necessitating large injection volumes to maintain sensitivity. However, the direct injection of an excessive amount of sample matrices poses a significant risk of carryover and accumulation in injector needles, valves, and the ion source. To mitigate this, a divert valve was integrated into the method. This setup directed the flow to the mass spectrometer only during the NDMA elution window (2.5–4.5 min), diverting early‐eluting salts and late‐eluting lipophilic matrix components to waste. This configuration effectively minimized source contamination and maintained long‐term instrument sensitivity.

### 3.4 Method Validation

The analytical method was validated in accordance with the ICH Q2(R2) guidelines “Validation of Analytical Procedures”, assessing specificity, LOD, LOQ, linearity, accuracy, precision, robustness, and system suitability [20].

Specificity was evaluated by confirming that the retention time of the analyte corresponded to the standard and by confirming the absence of interfering peaks in blank matrices. The acceptance criteria required analyte peak areas in blank samples to be ≤ 10% of the LOQ (2 ng/mL) and IS peaks to be ≤ 5%. While NDMA was detected in blank samples of ranitidine and nizatidine, the levels in nizatidine blanks slightly exceeded the acceptance criteria (Figure S2). However, given that the detected background signal remained close to the threshold and well below the regulatory limit for the drug product, this minor interference was deemed negligible and unlikely to compromise the accuracy of quantification. No interfering substances were observed at the retention time of the IS (Figure S3).

The LOD and LOQ were defined as the lowest analyte concentrations yielding S/N ratios of ≥ 3 and ≥ 10, respectively. Based on the S/N ratio of spiked samples, the LOD for NDMA was determined to be 1.0 ng/mL for most APIs, with a corresponding LOQ of 2.0 ng/mL. An exception was made for dutasteride, where the LOD and LOQ were established at 10 and 20 ng/mL, respectively. This higher quantification range is justified by the drug's low maximum daily dose (0.5 mg), which permits a higher allowable concentration of impurities while strictly adhering to the ADI threshold.

Linearity was assessed using standard solutions ranging from the LOQ to 100 ng/mL (2, 5, 10, 20, 50, and 100 ng/mL) for general APIs. For dutasteride, the linearity range was adjusted to 20–1000 ng/mL (20, 50, 100, 200, 500, and 1000 ng/mL). Calibration curves were constructed using the IS method (analyte/IS peak area ratio vs. concentration). In all cases, the coefficient of determination (*R* ^2^) exceeded 0.999 (Figure S4), satisfying the acceptance criterion of *R* ^2^ ≥ 0.990.

Accuracy and precision were evaluated by analyzing samples spiked with standard solutions at three concentration levels within the quantification range. Intra‐day and inter‐day accuracy and precision were assessed using five replicate measurements conducted on a single day and across five consecutive days, respectively. Intra‐day accuracy ranged from 87.7% to 115.5%, and inter‐day accuracy ranged from 89.4% to 114.1%, both falling within the acceptable limits (Table 2). The coefficients of variation (CV) for intra‐day and inter‐day precision were 0.17%–4.19% and 0.57%–10.65%, respectively, fully meeting the validation criteria.

**TABLE 2:** Intra‐day and inter‐day accuracy and precision for the quantification of NDMA across diverse pharmaceutical products. The method validation results are summarized for various commercial drug products representing a broad spectrum of active pharmaceutical ingredients (APIs) and complex excipient matrices. Accuracy (expressed as relative percentage) and precision (expressed as relative standard deviation, %RSD) were rigorously evaluated at three distinct theoretical concentration levels (5, 40, and 80 ng/mL) for all tested formulations. Intra‐day assessments were performed in triplicate (*n* = 3), while inter‐day assessments were conducted across five independent analytical runs (*n* = 5). The consistent recovery rates and low RSD values confirm the robust analytical reliability, high reproducibility, and universal applicability of the developed LC–APCI‐MS/MS method regardless of the specific drug matrix.

Pharmaceutical product	Theoretical conc. (ng/mL)	Intra‐day (*n* = 3)		Inter‐day (*n* = 5)	
		Accuracy (%)	Precision (%)	Accuracy (%)	Precision (%)
**Ranitidine**	5	104.6	0.3	104.4	2.0
	40	100.3	0.7	99.0	1.2
	80	100.3	0.2	99.7	0.8
**Nizatidine**	5	104.6	1.1	104.6	2.1
	40	100.3	1.4	99.0	1.2
	80	100.3	0.4	99.9	1.1
**Valsartan**	5	107.2	1.9	107.8	0.6
	40	100.0	0.9	98.9	1.1
	80	100.9	0.3	100.0	0.8
**Losartan**	5	99.7	2.6	102.1	2.5
	40	100.5	0.4	99.3	1.1
	80	101.8	1.1	98.1	3.4
**Olmesartan**	5	99.9	0.3	103.6	3.1
	40	102.0	0.6	100.6	1.3
	80	102.6	0.7	98.3	3.8
**Irbesartan**	5	98.5	1.5	102.4	3.3
	40	99.6	0.5	99.8	1.1
	80	104.5	0.6	101.6	2.5
**Candesartan**	5	100.2	0.7	105.5	4.4
	40	101.7	0.5	100.6	1.1
	80	102.8	1.0	98.1	4.3
**Fimasartan**	5	106.4	1.8	107.3	0.9
	40	100.2	0.7	99.0	1.1
	80	101.0	0.2	100.2	0.7
**Tramadol drug A**	5	93.3	1.7	92.7	1.6
	40	94.8	0.5	93.3	2.2
	80	94.9	0.7	94.9	3.6
**Tramadol drug B**	5	92.3	2.5	89.4	2.9
	40	93.4	2.7	93.7	1.9
	80	94.4	2.0	93.9	1.9
**Tramadol drug C**	5	92.0	4.1	90.4	1.8
	40	95.7	0.3	93.8	1.9
	80	96.6	0.8	94.6	4.3
**Tramadol drug D**	5	98.8	2.0	94.1	4.4
	40	102.6	1.2	97.4	5.1
	80	97.9	2.0	95.2	3.6
**Tramadol drug E**	5	97.4	1.9	92.0	5.1
	40	98.6	1.2	96.1	3.6
	80	106.0	2.5	99.5	5.9
**Tramadol drug F**	5	116.1	1.5	103.4	10.7
	40	104.6	0.6	98.9	5.2
	80	102.4	0.6	98.7	3.3
**Diltiazem drug A**	5	112.7	2.4	109.2	5.0
	40	101.2	0.6	100.7	1.6
	80	103.3	1.1	103.1	2.9
**Diltiazem drug B**	5	114.2	1.0	114.1	3.3
	40	104.1	2.4	103.1	3.7
	80	106.7	2.4	101.0	3.8
**Doxylamine**	5	115.5	2.0	112.1	2.7
	40	104.3	0.4	103.1	2.0
	80	102.8	0.8	101.0	3.1
**Rivastigmine**	5	106.3	1.2	107.1	2.4
	40	95.8	1.6	94.9	1.6
	80	100.6	1.7	97.9	2.7
**Metoclopramide**	5	94.7	2.9	100.1	5.2
	40	88.9	0.8	95.5	7.3
	80	97.5	1.2	100.5	3.6
**Entacapone**	5	108.6	2.4	107.4	7.3
	40	104.1	2.4	103.4	3.2
	80	103.4	0.9	101.2	1.9
**Prednisolone**	5	109.3	4.0	107.4	6.0
	40	99.6	1.6	101.9	2.1
	80	100.8	1.6	101.2	0.9
**Clarithromycin**	5	112.1	2.3	108.2	4.2
	40	99.7	2.1	100.8	1.1
	80	101.9	1.2	101.8	1.9
**Chlorpheniramine**	5	112.0	0.9	109.4	2.1
	40	104.4	1.6	103.1	1.5
	80	103.1	0.4	100.1	3.5
**Chlorpromazine**	5	114.5	0.9	110.3	6.0
	40	101.1	0.3	100.1	2.3
	80	100.9	1.1	99.5	1.4
**Imipramine**	5	98.8	0.9	103.9	4.3
	40	94.4	1.5	99.2	6.7
	80	94.0	4.2	97.3	6.1
**Amitriptyline**	5	102.9	3.6	106.2	3.7
	40	91.9	0.2	97.1	5.9
	80	91.4	2.0	96.2	6.3
**Desvenlafaxine**	5	101.5	4.0	108.2	7.9
	40	94.5	1.0	97.5	5.9
	80	87.7	0.9	92.6	8.3
**Dutasteride drug A**	5	106.0	1.0	103.1	2.9
	40	91.8	0.9	92.6	1.8
	80	94.4	1.5	95.1	1.3
**Dutasteride drug B**	5	109.8	0.8	112.2	3.3
	40	95.5	0.2	93.8	2.8
	80	94.1	1.8	93.9	1.4
**Dutasteride drug C**	5	102.7	0.7	100.1	4.9
	40	99.6	0.3	99.3	0.6
	80	93.0	1.5	93.5	1.3

Abbreviation: NDMA, *N*‐nitrosodimethylamine.

Robustness was examined to verify the method's capacity to withstand small, intentional variations in parameters. The flow rate and column oven temperature were altered by ±10%, and the resulting variations in peak area were monitored. All observed differences remained within 25% (Table S1), confirming the reliability of the method under routine operating conditions.

System suitability was verified by assessing the repeatability of the analyte‐to‐IS peak ratio at the LOQ level across six replicate injections. All CV values were maintained below 5% (Table S2), ensuring instrument performance.

### 3.5 Application to Pharmaceutical Products

The developed LC–APCI–MS/MS method was applied to determine NDMA content in a total of 30 commercially available pharmaceutical products. Among the tested samples, NDMA was clearly detected and quantified above the LOQ (2.0 ng/mL) in ranitidine, nizatidine, and amitriptyline products. The mean detected concentrations were 164.83 ng/mL for ranitidine, 10.25 ng/mL for nizatidine, and 2.28 ng/mL for the amitriptyline. Based on their respective maximum daily doses (300 mg/day for ranitidine and nizatidine products, and 150 mg/day for amitriptyline product), the estimated daily intakes of NDMA were approximately 1648 ng/day for ranitidine and 103 ng/day for nizatidine. Both of these values significantly exceeded the ADI limit of 96 ng/day established by regulatory agencies, while amitriptyline (approximately 23 ng/day) remained within the acceptable safety threshold. In contrast, for the remaining 27 products, including various sartans (valsartan, losartan, etc.), tramadol, and other APIs, NDMA was not detected.

It is important to note that the pharmaceutical products analyzed in this study were historical samples acquired in the past and subjected to prolonged storage prior to analysis. While the majority of these aged samples remained stable without any NDMA formation, the remarkably high NDMA levels observed in the histamine type 2 receptor antagonists (H2RAs; ranitidine and nizatidine) are highly consistent with their well‐documented degradation kinetics. Regulatory investigations have established that NDMA in these specific drug products is not solely a manufacturing impurity, but can generate and accumulate over time during storage, a process significantly accelerated by environmental factors such as moisture and ambient thermal stress [10, 18]. Similarly, the structurally vulnerable amitriptyline sample inherently contains a dimethylamine moiety, a well‐known direct precursor for NDMA. This structural vulnerability likely facilitated the gradual post‐manufacturing generation of NDMA through slow degradation or persistent interactions with nitrite‐containing excipients during its prolonged shelf life.

In contrast, for the remaining 27 products, including various sartans (valsartan, losartan, etc.), tramadol, and other active ingredients, NDMA was not detected. The comprehensive monitoring results are summarized in Table 3 and Table S3. Ultimately, the successful quantification of these storage‐induced NDMA fluctuations strongly demonstrates the method's practical utility and reliability for real‐world pharmaceutical surveillance.

**TABLE 3:** Monitoring results of NDMA in various commercially available pharmaceutical products using the proposed LC–MS/MS method.

**Therapeutic class**	**Active pharmaceutical ingredient (API)**	**No. of products**	**NDMA concentration (ng/mL)**
H2‐receptor antagonists	Ranitidine	1	164.83 ± 5.68
	Nizatidine	1	10.25 ± 0.52
Tricyclic antidepressants (TCAs)	Amitriptyline	1	2.28 ± 0.19
Angiotensin II receptor blockers (Sartans)	Valsartan, losartan, irbesartan, olmesartan, candesartan, fimasartan	6	N.D.^a^
Others	Imipramine, chlorpheniramine, metoclopramide, tramadol (A∼F), diltiazem (A∼B), Dutasteride (A∼C), etc.	21	N.D.^a^
Total		30	

^a^ N.D.: Not detected. Limit of quantitation (LOQ) for the method is 2.0 ng/mL (20 ng/mL for dutasteride).

Data are represented as mean ± SD (*n* = 3).

### 3.6 Comparison With Reported Methods

To benchmark the analytical performance of the developed method, we compared it with official regulatory protocols and representative published methodologies (Table 4). As highlighted by Alsohaimi et al. [16], detecting trace‐level carcinogens in complex pharmaceutical matrices presents significant analytical challenges. Consequently, methods established by regulatory agencies such as the FDA are well‐established but strictly drug specific. For instance, the official LC–HRMS methods necessitate distinct stationary phases—such as F5 (PFP) for sartans and C18 for ranitidine—imposing a significant operational burden by requiring frequent column switching and system re‐equilibration [38, 39].

**TABLE 4:** Comparison of the proposed LC–APCI–MS/MS method with previously reported and regulatory methods for the determination of NDMA.

**Method/reference**	**Target APIs**	**Analytical technique**	**Stationary phase**	**LOD/LOQ (ng/mL)**	**Key characteristics and limitations**
Official Methods (FDA) [38, 39]	Sartans, ranitidine	LC–HRMS/LC–MS/MS	F5, C18	0.03/1.0	*Characteristics*: Enables simultaneous determination of multiple nitrosamines.
					*Limitation*: Highly drug‐specific; necessitates frequent column switching for different therapeutic classes.
Gimenez‐Campillo et al. [3]	Ranitidine	GC‐MS	HP‐5MS	6.6/21 (ng/g)	*Characteristics*: Utilizes efficient microextraction for ranitidine analysis.
					*Limitation*: Risk of thermal degradation and artifact formation during GC analysis.
Masada et al. [4]	Valsartan	HPLC–DAD	C18	8.5/28.5	*Characteristics*: Cost‐effective analysis using optical detection.
					*Limitation*: Requires multiple extraction steps and precludes the use of stable isotope‐labeled internal standards (SIL‐IS); limited to valsartan.
Lim et al. [5]	Sartans, ranitidine, metformin	GC‐MS/MS	DB‐WAX	0.3/0.9 (ng/g)	*Characteristics*: Eliminate GC thermal artifact formation by completely removing APIs in sample preparation step.
					*Limitation*: Requires highly labor‐intensive and time‐consuming sample preparation (precipitation combined with SPE), significantly reducing analytical throughput.
Liu et al. [24]	Ranitidine	LC–MS/MS	C18	1.0/3.0	*Characteristics*: Utilizes a 10‐way switching valve to prevent MS contamination from the API.
					*Limitation*: The valve‐switching timings are strictly hard‐coded to ranitidine, making the setup highly matrix‐dependent and difficult to adapt for diverse drugs.
Malihi et al. [26]	Ophthalmic solutions	LC–MS/MS	PFP	0.06/0.2	*Characteristics*: Highly sensitive; optimized for specific liquid formulations; enables simultaneous determination of multiple nitrosamines.
					*Limitation*: Validated only for specific liquid matrix; lacks universal applicability across diverse and complex solid oral dosage forms.
Proposed method	22 diverse APIs (ARBs, ranitidine, etc.)	LC–MS/MS	C30	1.0/2.0 (10/20 for dutasteride)	*Characteristics*: Universal platform; single setup eliminates drug‐specific method development; robust against thermal artifacts and matrix effects.
					*Limitation*: Method validation is currently restricted to a single nitrosamine (NDMA).

Similarly, many reported NDMA methods are highly tailored to a single therapeutic class or matrix. Masada et al. [4] developed an HPLC–DAD method specifically for valsartan, but its reliance on optical detection necessitates multiple complex extraction steps and precludes the use of stable isotope‐labeled internal standards (SIL‐IS), which compromises quantitative reliability. Furthermore, while Liu et al. [24] utilized an LC–MS/MS approach for ranitidine, the insufficient retention of polar NDMA on conventional C18 columns compelled the use of a complex 10‐way valve‐switching technique as a primary means of separation to physically divert the massive API peak. This rigid hardware setup makes their method strictly matrix‐dependent and difficult to adapt for other drugs without re‐optimization.

Attempts to overcome this fragmentation through multi‐class methods have faced other critical hurdles. The GC–MS/MS approach proposed by Lim et al. [5] aimed to analyze sartans, metformin, and ranitidine simultaneously. However, to circumvent the well‐documented issue of thermal artifact formation during the GC analysis of unstable APIs like ranitidine [12, 13], their method required highly labor‐intensive and time‐consuming sample preparation involving both precipitation and solid‐phase extraction (SPE), which significantly reduces analytical throughput.

In contrast, the proposed method leverages the superior shape selectivity and retention of the C30 stationary phase for polar nitrosamines. As summarized in Table 4, this approach achieves adequate chromatographic separation of NDMA from 30 diverse APIs, entirely eliminating the reliance on complicated hardware modifications or labor‐intensive SPE procedures. While we also incorporated a switching valve, its function is primarily for MS source protection rather than a mandatory separation tool, allowing the platform to remain universal. Achieving an LOD and LOQ of 1.0 and 2.0 ng/mL (for most APIs), respectively—which are fully adequate for regulatory compliance—this approach enables universal determination using a simple “dilute‐and‐shoot” preparation. Although a recognized limitation is that the current validation is restricted to NDMA, the unique properties of the C30 phase provide a robust foundation for expanding this platform to other nitrosamine impurities in future studies. Ultimately, this universality significantly streamlines the quality control workflow, enhancing throughput and reliability for pharmaceutical laboratories handling diverse product lines.

## 4 Conclusions

This study developed and validated a novel, universal LC–APCI–MS/MS method for the sensitive and selective quantification of NDMA across 30 diverse pharmaceutical products. Unlike existing drug‐specific methods that require separate, resource‐intensive protocols for each drug owing to their varied physicochemical properties, this universal approach simplifies the analytical workflow. Specifically, this method leverages the superior shape selectivity of the C30 stationary phase and an optimized mobile phase to achieve effective chromatographic resolution of NDMA from massive and diverse API substances. In addition, a divert valve is strategically integrated to protect the mass spectrometer from high‐concentration API matrices, ensuring long‐term system robustness without compromising analytical universality.

Our monitoring results revealed that, despite all products being historical batches subjected to prolonged storage, H2RAs were the most contaminated. Specifically, ranitidine exhibited the highest NDMA levels (164.98 ± 5.68 ng/mL), followed by nizatidine (10.25 ± 0.52 ng/mL), both of which significantly exceeded the regulatory ADI limits. Meanwhile, amitriptyline showed detectable levels (2.28 ± 0.19 ng/mL) strictly within the safety threshold. Furthermore, sartans and all other 27 tested drugs showed no detectable NDMA. Crucially, the detection of these impurities in these historical samples highlights the severe risk of post‐manufacturing NDMA generation during prolonged storage. This gradual accumulation is driven by environmental degradation and inherent structural vulnerabilities, such as the dimethylamine moiety present in amitriptyline.

A limitation of the current study is that full validation was restricted to NDMA and did not encompass other nitrosamine impurities such as *N*‐nitrosodiethylamine (NDEA), *N*‐nitrosomethylphenylamine (NMPA), or *N*‐nitrosoethylmethylamine (NEMA). However, given the superior shape selectivity of the C30 stationary phase toward planar and isomeric molecules, the method holds significant theoretical potential for the simultaneous separation of these structurally related compounds [29, 30, 33]. Furthermore, the APCI source utilized in this study is highly effective for ionizing these volatile and less polar nitrosamines. Future work will focus on expanding the method's scope to include these impurities and evaluating its applicability to complex matrices such as environmental water and biological fluids, as emphasized in recent reviews [11].

Based on these findings, we suggest the adoption of this universal C30‐based platform for routine regulatory screening to proactively manage the risk of storage‐induced nitrosamine contamination. Ultimately, this innovative approach establishes a sustainable, reproducible, and reliable platform for NDMA analysis, strictly adhering to ICH Q2(R2) guidelines. Its simplicity, robustness, and broad applicability make it a pivotal tool for pharmaceutical companies and regulatory agencies. By serving as an efficient screening solution across diverse drug formulations, this method streamlines method transfer, enhances regulatory compliance, and safeguards product quality to ensure patient safety. This research significantly advances nitrosamine impurity analysis, addressing the urgent need for a universal monitoring method to protect the pharmaceutical supply chain.

## Author Contributions

**Byungchan An**: conceptualization, methodology, validation, formal analysis, data curation, writing – original draft. **Unyong Kim**: data curation, investigation, formal analysis, writing – review and editing. **Sumin Seo**: data curation, visualization. **Jiyu Kim**: formal analysis, investigation. **Chohee Jeong**: investigation, visualization. **Woojin Jeong**: formal analysis, investigation. **Eunjin Ko**: investigation, formal analysis. **Juhyeon Kim**: methodology, investigation. **Hyun‐Deok Cho**: investigation, formal analysis. **Sang Beom Han**: conceptualization, supervision, resources, funding acquisition, investigation, methodology, writing – review and editing, project administration.

## Funding

This research was supported by grants (20173MFDS162 and 23194MFDS086) from the Ministry of Food and Drug Safety in 2023, and by the Ministry of Education (Grant Number: 2021R1A6A1A03044296).

## Conflicts of Interest

The authors declare no conflicts of interest.

## Supporting information




**Supporting File**: jssc70465‐sup‐0001‐SuppMat.docx.

## Data Availability

The data that support the findings of this study are available from the corresponding author upon reasonable request.
