# Supplementary material for: A Robust and Universal LC–MS/MS Method for Determination of N‐Nitrosodimethylamine in Pharmaceuticals Using a C30 Column
Source: J Sep Sci. 2026 Jun 12;49(6):e70465. doi: 10.1002/jssc.70465 (PMC13261539; doi:10.1002/jssc.70465)
Supplement: Supplementary file 1 — Supporting File: jssc70465‐sup‐0001‐SuppMat.docx. [file JSSC-49-e70465-s001.docx]

**Supplementary Materials**

**A robust and universal LC-MS/MS method for determination of *N*-nitrosodimethylamine in pharmaceuticals using a C30 column**

Byungchan An^a, ⁋^, Unyong Kim^a, ⁋^, Sumin Seo^a^, Jiyu Kim^a^, Chohee Jeong^a^, Woojin Jeong^a^, Eunjin Ko^a^, Juhyeon Kim^a^, Hyun-Deok Cho^b^, Sang Beom Han^a,^ *

*^a^Department of Pharmaceutical Analysis, College of Pharmacy, Chung-Ang University, 84 Heukseok-ro, Dongjak-gu, Seoul 06974, Republic of Korea*

*^b^Division of Non-clinic Studies, Korea Institute of Toxicology, 141 Gajeong-ro, Yuseong-gu, Daejeon, 34114, Republic of Korea*

^⁋^These authors contributed equally to this work.

* Corresponding Author:

Sang Beom Han, Ph.D.

Department of Pharmaceutical Analysis

College of Pharmacy, Chung-Ang University

84 Heukseok-ro, Dongjak-gu

Seoul 06974, Republic of Korea

Tel: +82-2-820-5596

E-mail: hansb@cau.ac.kr

**
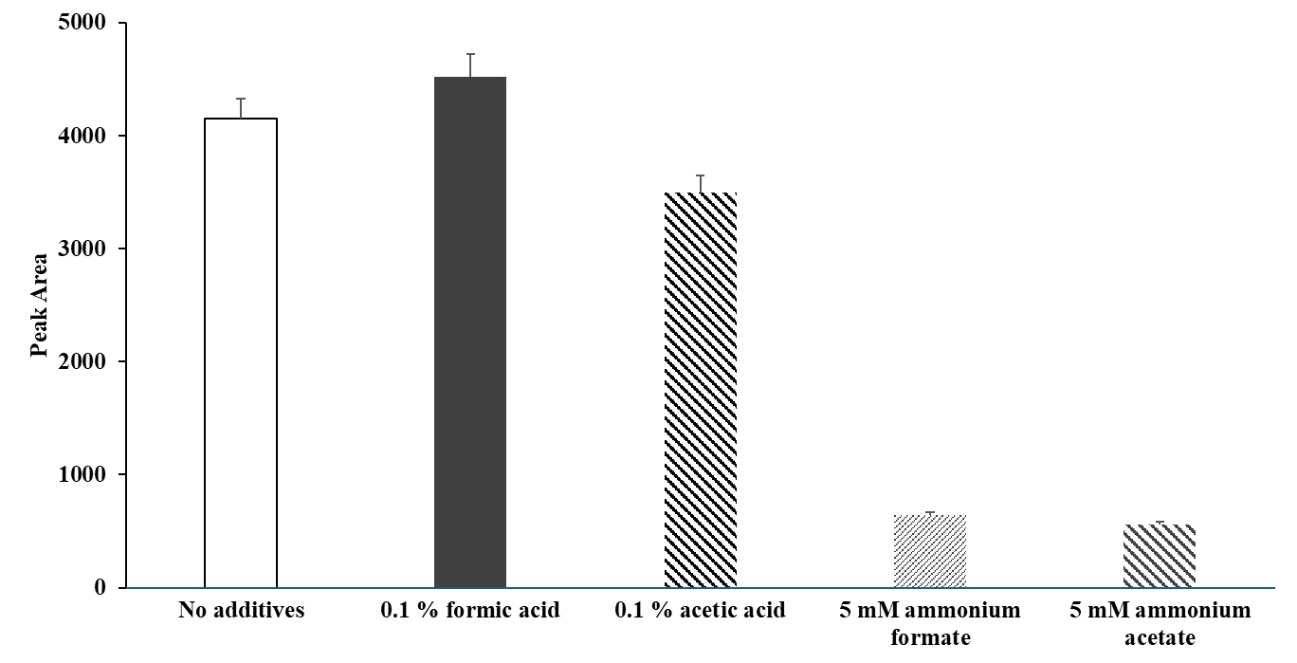
**

**Fig. S1.** **Effect of different aqueous mobile phase additives on the peak area of NDMA in LC-APCI-MS/MS analysis.** The organic solvent used was methanol. Data represent the mean ± standard deviation (n = 3). The use of 0.1% v/v formic acid yielded the highest sensitivity compared to other additives (acetic acid, ammonium formate, ammonium acetate) or no additive. *Conditions:* Develosil RPAQUEOUS-AR C30 column; Flow rate: 1.0 mL/min; Column temperature: 30 °C.

**Fig. S2**

**
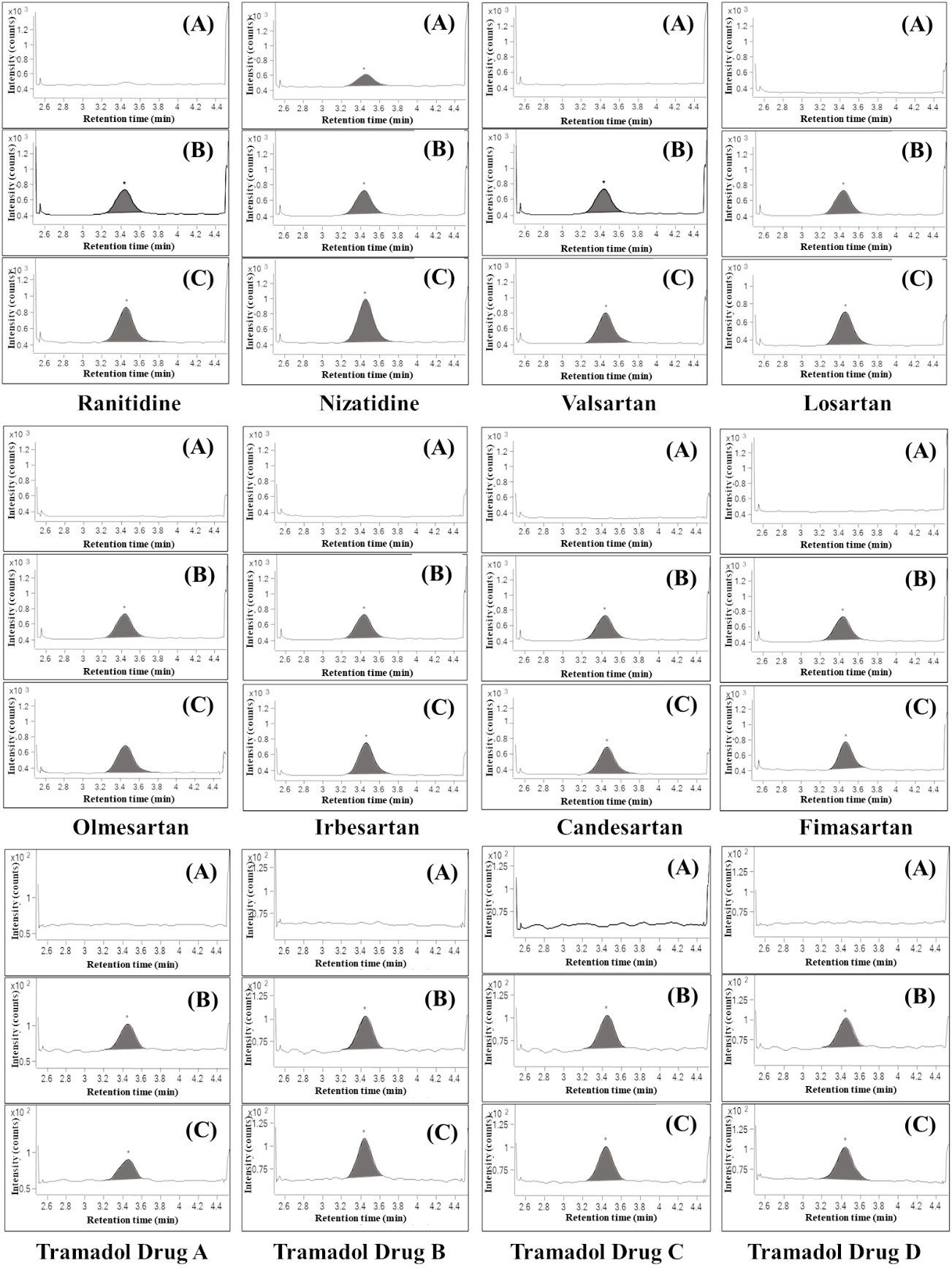
**

**Fig. S2 (continued)**

**
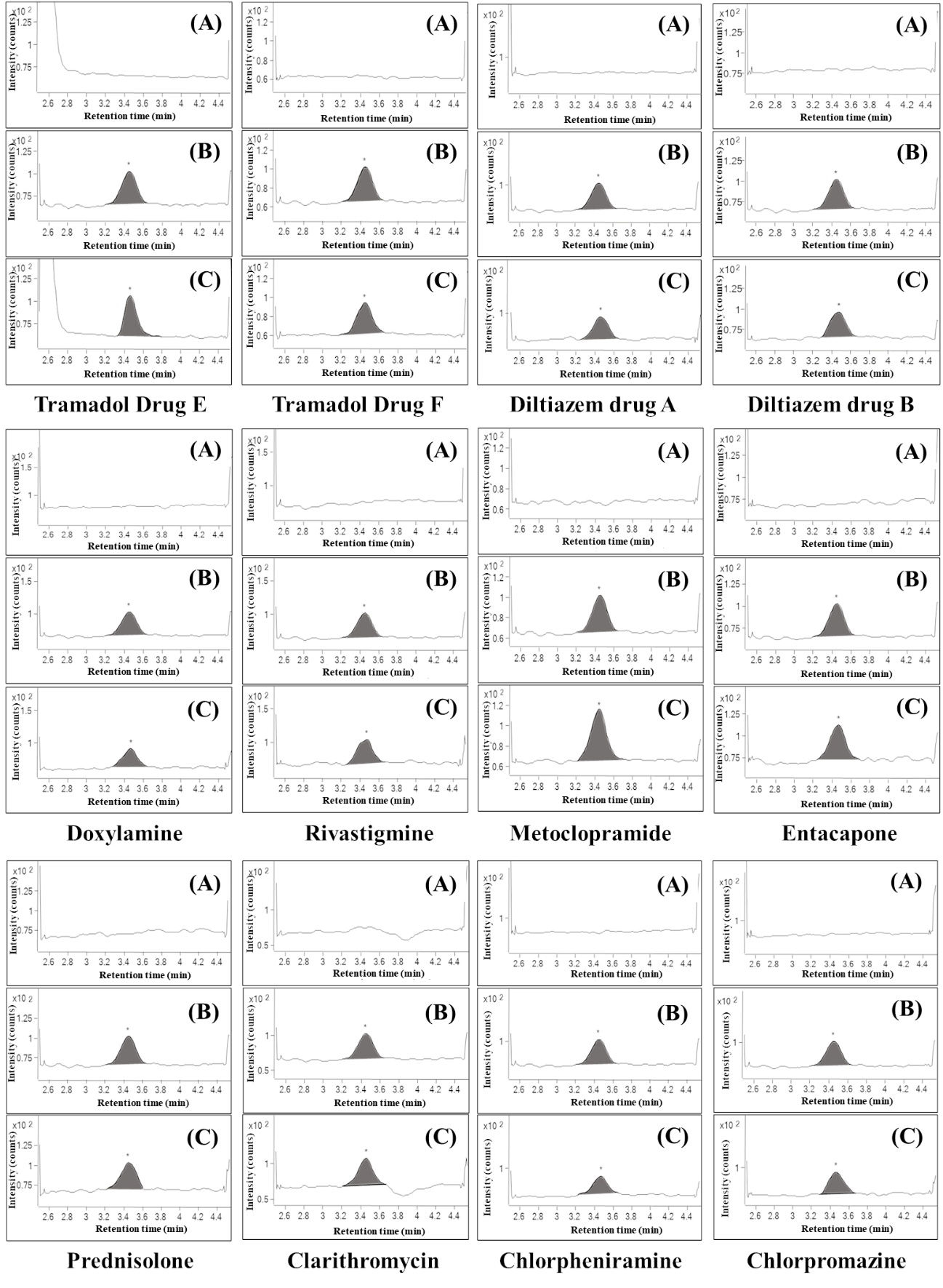
**

**Fig. S2 (continued).**

**
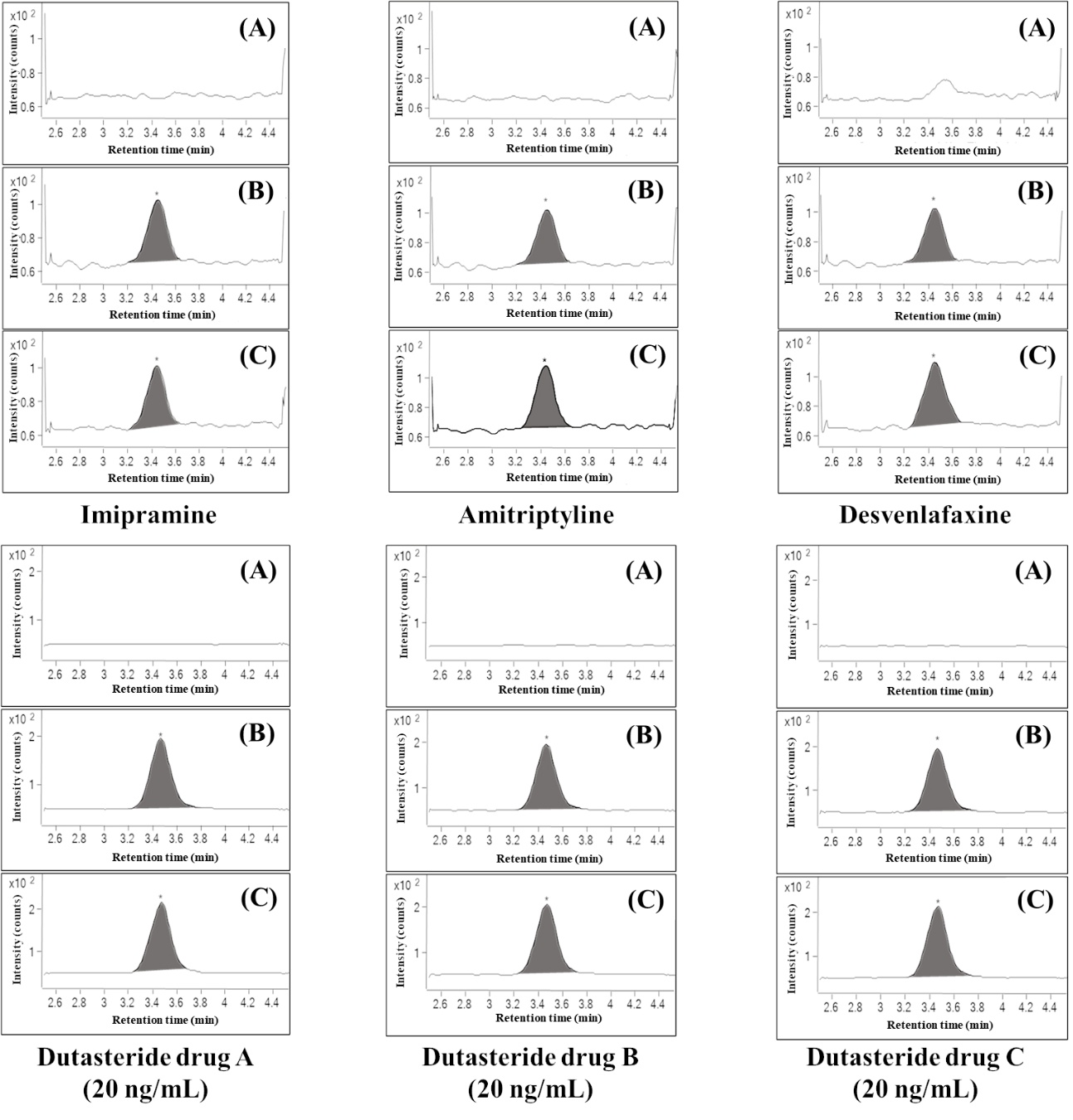
**

**Fig. S2.** **Representative LC-APCI-MS/MS multiple reaction monitoring (MRM) chromatograms of NDMA for the specificity assessment across 22 diverse pharmaceutical products.** The panels display chromatograms for (A) blank matrix samples, (B) NDMA standard solutions at the limit of quantification (LOQ, 2.0 ng/mL), and (C) sample matrices spiked with NDMA at the LOQ level (2.0 ng/mL). The chromatograms confirm the absence of significant matrix interferences at the retention time of NDMA, demonstrating the excellent selectivity of the C30 stationary phase for all tested active pharmaceutical ingredients (APIs).

**Fig. S3**

**
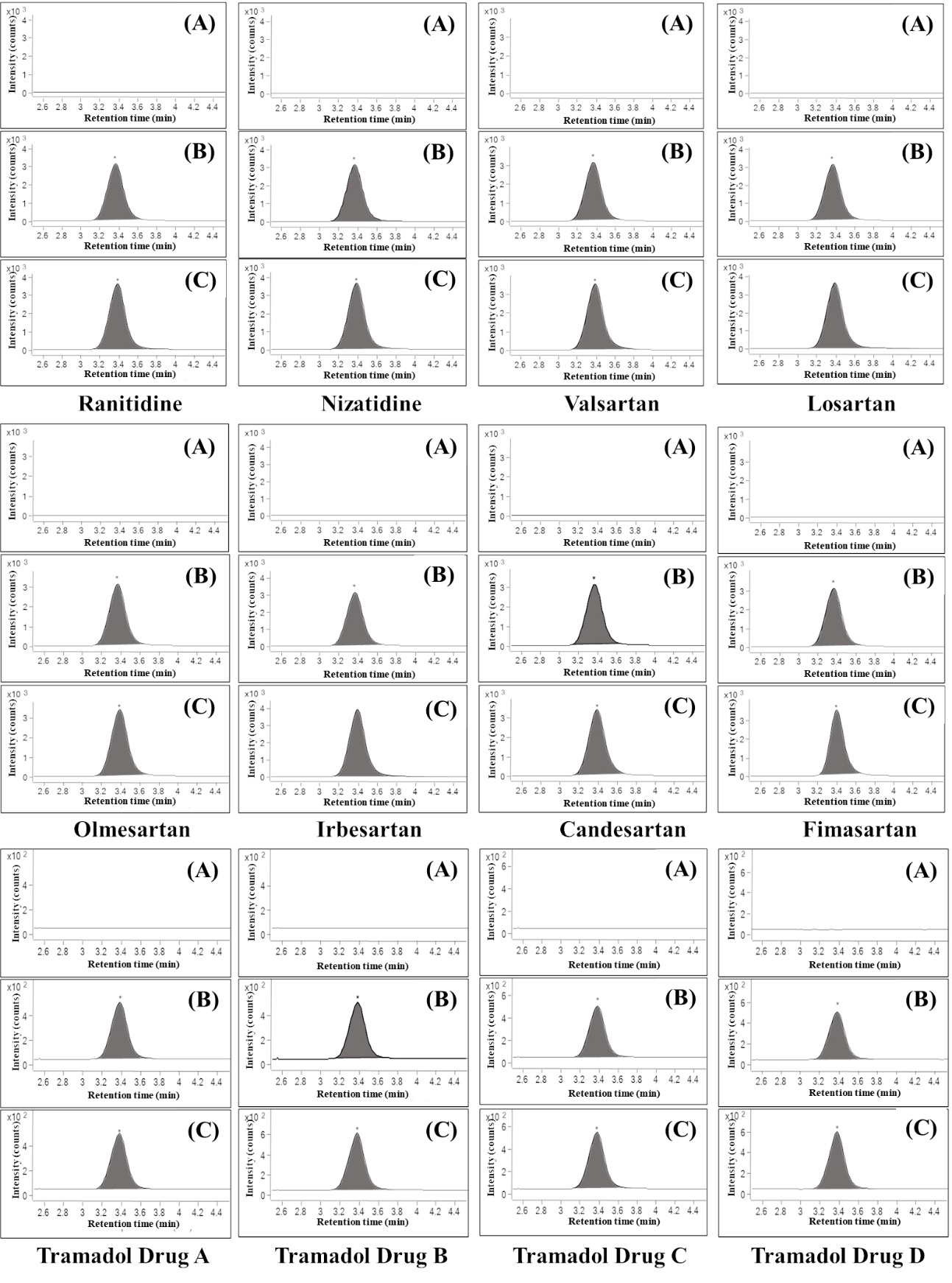
**

**Fig. S3 (continued)**

**
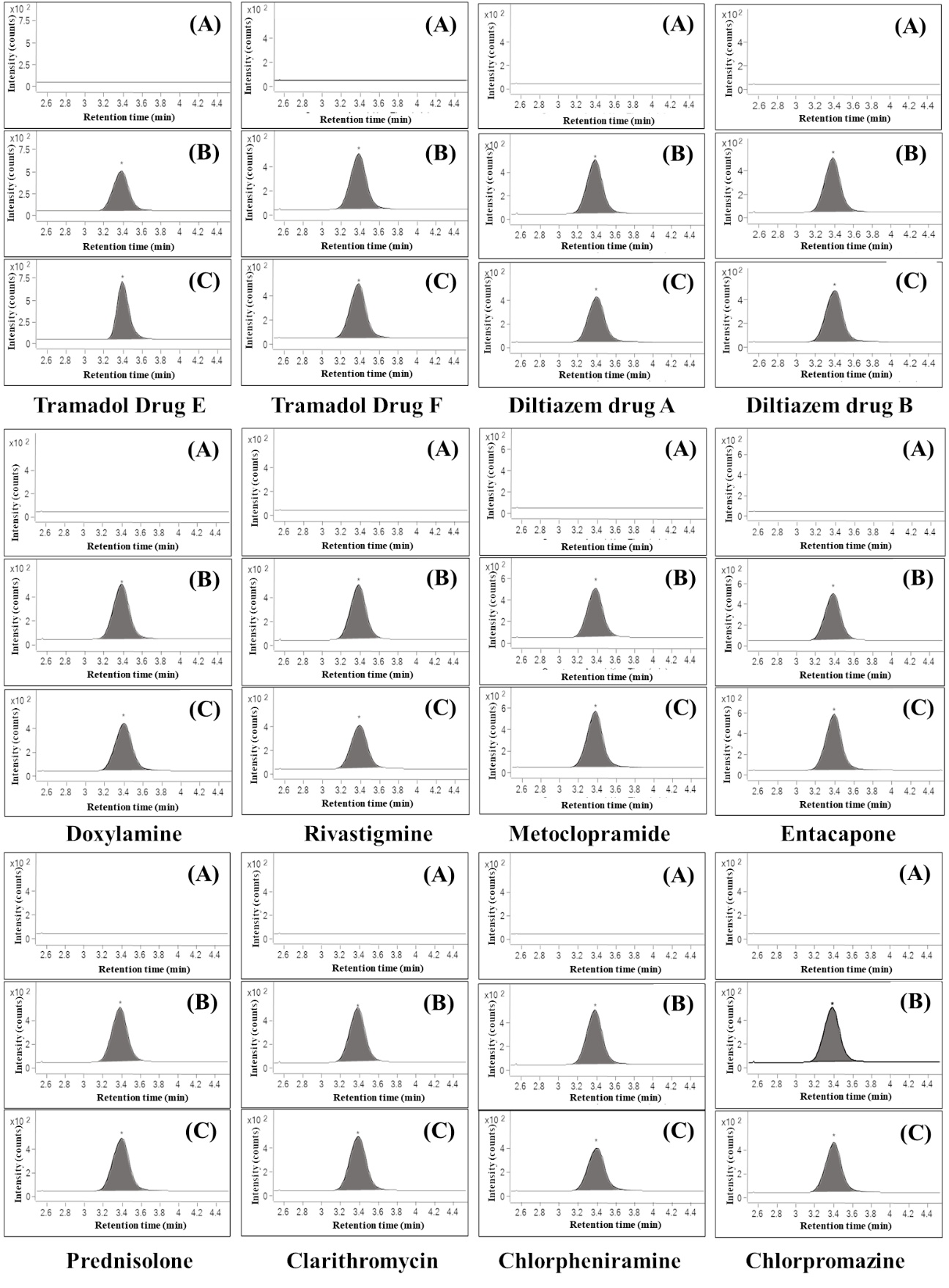
**

**Fig. S3 (continued)**

**
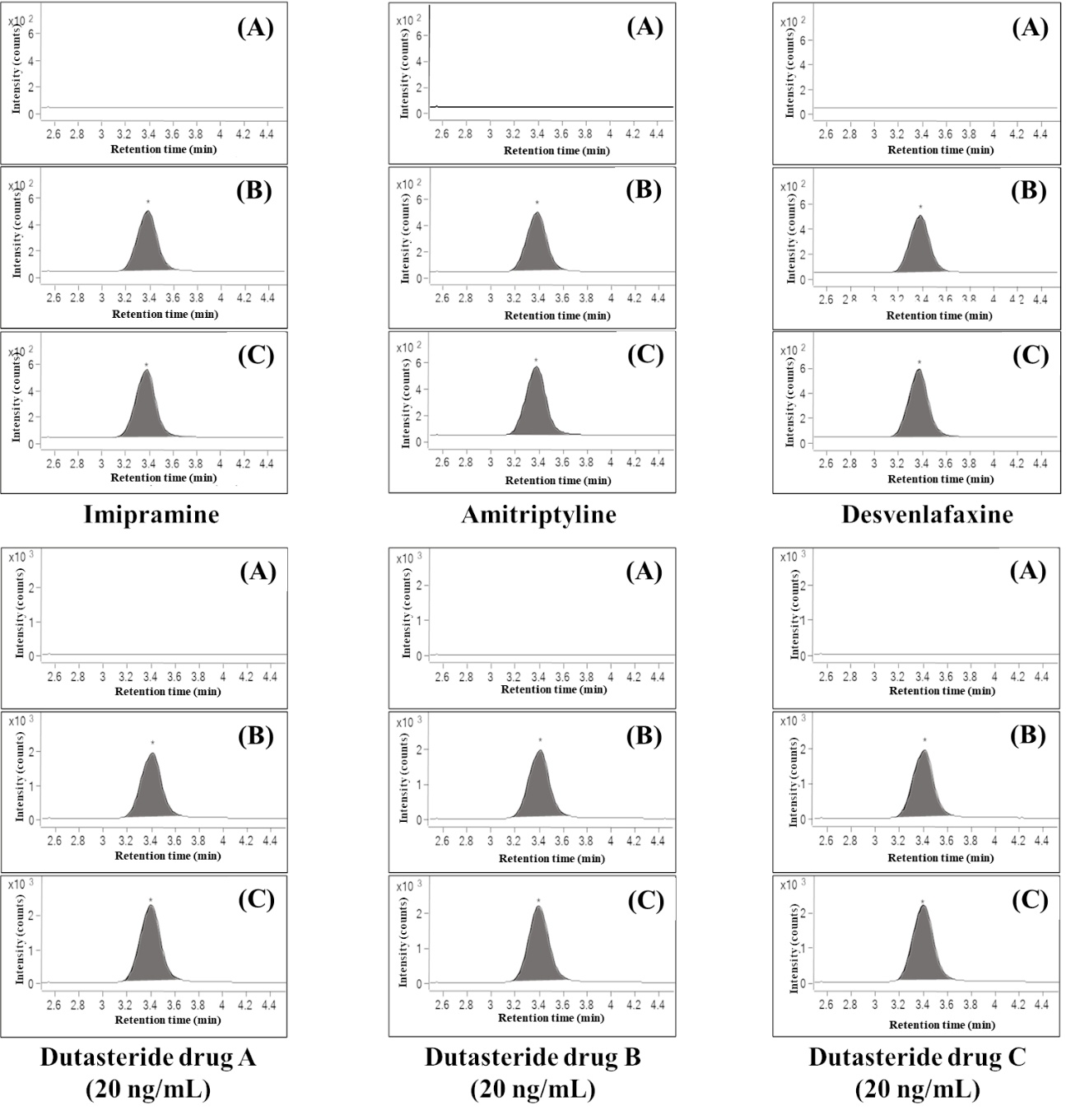
**

**Fig. S3.** **Representative LC-APCI-MS/MS multiple reaction monitoring (MRM) chromatograms of the stable isotope-labelled internal standard (SIL-IS, NDMA-d6), across 22 diverse pharmaceutical products.** The panels display chromatograms for (A) blank matrix samples without the internal standard, (B) NDMA-d6 standard solutions (20 ng/mL), and (C) sample matrices spiked with NDMA-d6 (20 ng/mL). The results verify that there are no co-eluting endogenous compounds or matrix peaks interfering with the quantification of the internal standard at its specific retention time.

**
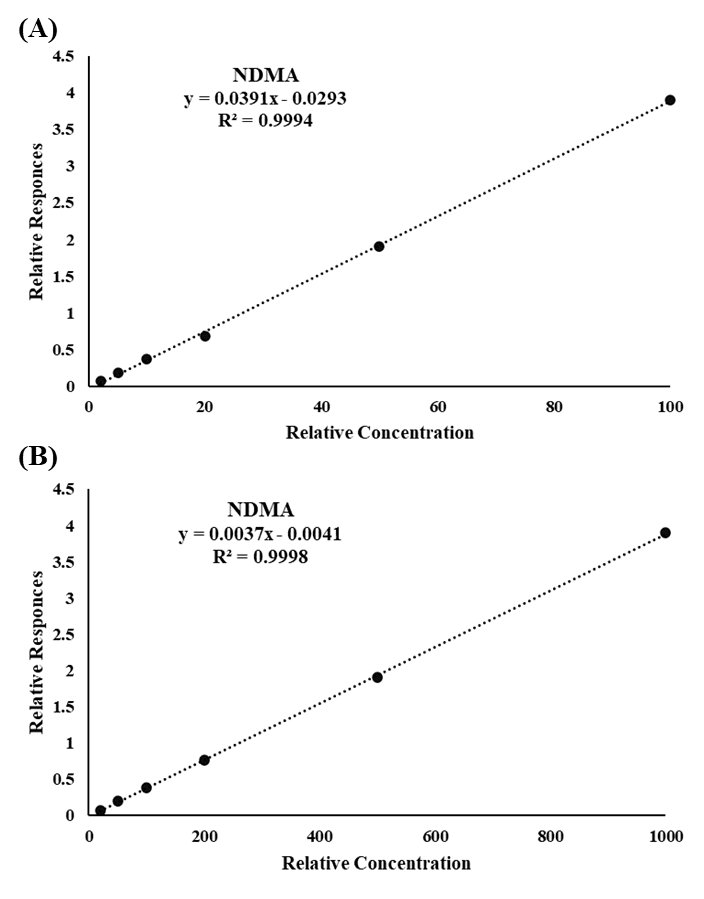
**

**Fig. S4. Calibration curves for NDMA using the internal standard (IS) method. The graphs plot the relative response (peak area ratio of NDMA to NDMA-d6) against the relative concentration.** (A) Linear dynamic range from 2.0 to 100.0 ng/mL, applied to most of the evaluated pharmaceutical products. (B) Extended linear dynamic range from 20.0 to 1000.0 ng/mL, specifically optimized and applied for dutasteride formulations due to their lower maximum daily dose. Both curves exhibit excellent linearity with coefficients of determination (*R*^2^) greater than 0.999.

**Supplementary Tables**

**Table S1.** **Robustness assessment of the developed LC-APCI-MS/MS method.** The table summarizes the effect of internal, minor variations in key chromatographic parameters-specifically flow rate (±10%, 0.9 to 1.1 mL/min) and column oven temperature (±10%, 27 to 33 °C)-on the retention time (RT) and peak area of NDMA and the internal standard (NDMA-d6). The rate of change (ROC) for the peak area ratio (STD/IS) remained well within acceptable limits, confirming the method’s reliability under routine operational fluctuations.

| **Parameters** | **Conditions** | **NDMA** | | | | **NDMA-d6** | | | | **STD/IS** | |
| --- | --- | --- | --- | --- | --- | --- | --- | --- | --- | --- | --- |
|  |  | **RT***  **(min)** | **ROC* (%)** | **Peak area** | **ROC* (%)** | **RT***  **(min)** | **ROC* (%)** | **Peak area** | **ROC* (%)** | **Peak area ratio** | **ROC* (%)** |
| **Flow rate**  **(mL/min)** | 0.9 | 3.8 | 11.2 | 1108 | 2.0 | 3.8 | 11.4 | 6480 | 10.2 | 0.17 | -7.4 |
|  | 1.0 | 3.4 | - | 1086 | - | 3.4 | - | 5883 | - | 0.19 | - |
|  | 1.1 | 3.1 | -8.9 | 1223 | 12.6 | 3.1 | -9.0 | 6905 | 17.4 | 0.18 | -4.1 |
| **Column temperature (℃)** | 27 | 3.5 | 2.1 | 1285 | 18.3 | 3.5 | 2.1 | 7070 | 20.2 | 0.18 | -1.5 |
|  | 30 | 3.4 | - | 1086 | - | 3.4 | - | 5883 | - | 0.19 | - |
|  | 33 | 3.4 | -1.8 | 1159 | 6.7 | 3.3 | -1.8 | 6404 | 8.9 | 0.18 | -2.0 |

*RT: Retention time

*ROC: Rate of change

STD, standard; IS, internal standard

**Table S2.** **System suitability testing results demonstrating injection repeatability.** The data represent six replicate injections of the standard solution at the limit of quantification (LOQ) level. The precision is expressed as the coefficient of variation (CV, %) for the retention times and peak areas of both NDMA and NDMA-d6, as well as their peak area ratio (STD/IS). All CV values were maintained below 5%, confirming the suitability of the chromatographic system and analytical reproducibility prior to sample analysis.

| **Number of replicates** | **NDMA** | | **NDMA-d6** | | **The ratio of peak area (STD/IS)** |
| --- | --- | --- | --- | --- | --- |
|  | **Retention time** | **Peak area** | **Retention time** | **Peak area** |  |
| **1** | 3.47 | 5652 | 3.41 | 29499 | 0.19 |
| **2** | 3.48 | 5426 | 3.42 | 27212 | 0.20 |
| **3** | 3.47 | 5708 | 3.40 | 29927 | 0.19 |
| **4** | 3.48 | 5945 | 3.41 | 29603 | 0.20 |
| **5** | 3.47 | 5665 | 3.41 | 28363 | 0.20 |
| **6** | 3.47 | 5248 | 3.40 | 27413 | 0.19 |
| **Average** | 3.47 | 5607 | 3.41 | 28670 | 0.20 |
| **SD** | 0.01 | 241.4 | 0.01 | 1178.0 | 0.01 |
| **CV (%)** | 0.15 | 4.31 | 0.22 | 4.11 | 2.81 |

STD, standard; IS, internal standard; SD, standard deviation; CV, coefficient of variation

**Table S3. Detailed monitoring results of NDMA in 30 commercially available pharmaceutical products (n=3).**

| **No.** | **API / Drug Product** | **NDMA Concentration (ng/mL)** |
| --- | --- | --- |
| 1 | Ranitidine | 164.83 ± 5.68 |
| 2 | Nizatidine | 10.25 ± 0.52 |
| 3 | Amitriptyline | 2.28 ± 0.19 |
| 4 | Imipramine | N.D. |
| 5 | Chlorpheniramine | N.D. |
| 6 | Metoclopramide | N.D. |
| 7 | Entacapone | N.D. |
| 8 | Tramadol Product F | N.D. |
| 9 | Tramadol Product E | N.D. |
| 10 | Tramadol Product D | N.D. |
| 11 | Tramadol Product C | N.D. |
| 12 | Tramadol Product B | N.D. |
| 13 | Tramadol Product A | N.D. |
| 14 | Rivastigmine | N.D. |
| 15 | Prednisolone | N.D. |
| 16 | Olmesartan | N.D. |
| 17 | Irbesartan | N.D. |
| 18 | Losartan | N.D. |
| 19 | Candesartan | N.D. |
| 20 | Fimasartan | N.D. |
| 21 | Dutasteride Product C | N.D. |
| 22 | Dutasteride Product B | N.D. |
| 23 | Dutasteride Product A | N.D. |
| 24 | Doxylamine | N.D. |
| 25 | Diltiazem Product B | N.D. |
| 26 | Diltiazem Product A | N.D. |
| 27 | Desvenlafaxine | N.D. |
| 28 | Clarithromycin | N.D. |
| 29 | Chlorpromazine | N.D. |
| 30 | Valsartan | N.D. |

*N.D.: Not detected. Limit of quantitation (LOQ) for the method is 2.0 ng/mL (20 ng/mL for dutasteride).* *Values are expressed as the mean ± standard deviation of three independent measurements.*
